# In Silico Modeling and Structural Analysis of Soluble Epoxide Hydrolase Inhibitors for Enhanced Therapeutic Design

**DOI:** 10.3390/molecules28176379

**Published:** 2023-08-31

**Authors:** Shuvam Sar, Soumya Mitra, Parthasarathi Panda, Subhash C. Mandal, Nilanjan Ghosh, Amit Kumar Halder, Maria Natalia D. S. Cordeiro

**Affiliations:** 1 Department of Pharmaceutical Technology, Jadavpur University, Kolkata 700032, India; itzshuvam@gmail.com (S.S.);; 2 Dr. B. C. Roy College of Pharmacy and Allied Health Sciences, Campus Dr. Meghnad Saha Sarani, Durgapur 713206, India; 3 LAQV@REQUIMTE—Department of Chemistry and Biochemistry, Faculty of Sciences, University of Porto, 4169-007 Porto, Portugal

**Keywords:** soluble epoxide hydrolase, QSAR, feature selection, pharmacophore, molecular dynamics, Transformer-CNN

## Abstract

Human soluble epoxide hydrolase (sEH), a dual-functioning homodimeric enzyme with hydrolase and phosphatase activities, is known for its pivotal role in the hydrolysis of epoxyeicosatrienoic acids. Inhibitors targeting sEH have shown promising potential in the treatment of various life-threatening diseases. In this study, we employed a range of in silico modeling approaches to investigate a diverse dataset of structurally distinct sEH inhibitors. Our primary aim was to develop predictive and validated models while gaining insights into the structural requirements necessary for achieving higher inhibitory potential. To accomplish this, we initially calculated molecular descriptors using nine different descriptor-calculating tools, coupled with stochastic and non-stochastic feature selection strategies, to identify the most statistically significant linear 2D-QSAR model. The resulting model highlighted the critical roles played by topological characteristics, 2D pharmacophore features, and specific physicochemical properties in enhancing inhibitory potential. In addition to conventional 2D-QSAR modeling, we implemented the Transformer-CNN methodology to develop QSAR models, enabling us to obtain structural interpretations based on the Layer-wise Relevance Propagation (LRP) algorithm. Moreover, a comprehensive 3D-QSAR analysis provided additional insights into the structural requirements of these compounds as potent sEH inhibitors. To validate the findings from the QSAR modeling studies, we performed molecular dynamics (MD) simulations using selected compounds from the dataset. The simulation results offered crucial insights into receptor–ligand interactions, supporting the predictions obtained from the QSAR models. Collectively, our work serves as an essential guideline for the rational design of novel sEH inhibitors with enhanced therapeutic potential. Importantly, all the in silico studies were performed using open-access tools to ensure reproducibility and accessibility.

## 1. Introduction

Epoxide hydrolases are a family of widely distributed enzymes responsible for the rapid hydrolysis of epoxides into corresponding vicinal diols. The soluble epoxide hydrolase (sEH) is found in all lower and upper vertebrates, but only the mammalian sEH is associated with phosphatase activity [1,2]. Human soluble epoxide hydrolase (sEH) is a dual-functioning homodimeric enzyme and a member of the epoxide hydrolase family. It is involved in the hydrolysis of epoxyeicosatrienoic acids (EETs) [3,4]. Human sEHs are present in both cytosol and peroxisomes and exhibit hydrolase and phosphatase activities. In the presence of this enzyme, the biological effects of EETs are diminished. EETs are involved in various biological processes, including vasodilation of coronary arterioles, vascular smooth muscle relaxation, renal excretion of sodium, reduction of the expression of cytokine-induced endothelial cell adhesion molecules, and lipid and carbohydrate metabolism, as well as insulin resistance [5,6,7,8]. Additionally, EETs may contribute to neovascularization by promoting angiogenesis [8]. Consequently, sEH is responsible for degrading EETs into inactive products, thereby diminishing several protective mechanisms elicited by EETs. Inhibitors of sEH may then have implications in the treatment of various diseases such as diabetes, fibrosis, chronic pain, cardiovascular diseases, and neurodegenerative diseases [9]. These inhibitors are also claimed to be useful in the treatment of disorders related to smooth muscles, such as erectile dysfunction, hyperactive bladder, uterine contractions, irritable bowel syndrome (IBS), rheumatoid arthritis, and nephropathy [5,10]. Yet, the role of sEH mentioned above is primarily regulated through catalysis that occurs at the C-terminal hydrolase domain of the enzyme. The role of the N-terminal phosphatase domain has been comparatively less investigated, but there is strong evidence that the phosphatase activity of this domain is capable of hydrolyzing diverse lipid phosphates, including farnesyl pyrophosphate, sphingosine-1-phosphate, and lysophosphatidic acid. Recent studies have reported that inhibiting the phosphatase activity of sEH may prevent obesity and cardiac ischemic injury [11,12,13]. Several compounds inhibiting sEH functionalities have been reported, and some of these (e.g., SMTP-7, an investigational thrombolytic drug for the treatment of ischemic stroke; and ebselen, an anti-inflammatory, antioxidant, and cytoprotective drug) may simultaneously block both hydrolase and phosphatase activities [14]. Recently, it was discovered that sEH inhibition leads to a reduction in hepatic fat accumulation and inflammation, also suggesting a promising role in the treatment of Nonalcoholic Steatohepatitis (NASH) [15,16].

Researchers from the Goethe University, Germany, have been involved in the design and development of sEH inhibitors with a range of structural scaffolds [4,10,14]. The objective of this work is to perform a ligand-based in silico study utilizing receptor-independent Quantitative Structure-Activity Relationship (QSAR) modeling. The aim is to gain an understanding of the structural requirements of 184 compounds that have been reported by the researchers involved in such a study. QSAR, which is one of the oldest but most reliable in silico techniques, provides a viable option to minimize experimental work and screen novel molecules during drug design and development [17,18]. Whole molecular descriptor-based QSAR is particularly helpful in estimating the structural requirements for a diverse set of ligands with multiple mechanisms of action [19,20,21]. In recent years, QSAR methodologies have advanced with the discovery of various novel descriptors, and model-building strategies have also improved with the progress in feature selection methodologies and machine learning techniques coupled with computational efficiency [21,22]. As mentioned earlier, compounds inhibiting sEH may have multiple binding sites (hydrolase catalytic site and phosphatase catalytic site). Therefore, in this study, we primarily relied on QSAR regression methods to determine if validated predictive models can be generated with a dataset containing diverse sEH inhibitors that likely possess multiple binding mechanisms.

## 2. Results

### 2.1. 2D-QSAR Model

Following the strategy mentioned in Materials and Methods, we systematically sought the best linear 2D-QSAR MLR models. As mentioned, this involved using nine types of descriptors and two feature selection strategies, resulting in a total of 81 models. The outcomes of the 81 models are summarized in Table S3 of the Supplementary Materials. It was observed that each set of descriptors was capable of producing at least one model with acceptable Q^2^_LOO_ (>0.65) and R^2^_Pred_ (>0.50) values [23,24]. However, the primary objective was to identify the most predictive model in terms of statistical quality. It was found that the AlvaDesc descriptors along with the GA feature selection approach yielded the most successful model (Q^2^_LOO_ = 0.784 and R^2^_Pred_ = 0.792). The resulting model (an eight-variable equation) is given below together with the statistical parameters of the regression.

pIC50 (M) = +1.255(±0.296) + 1.542(±0.227) ATS6m − 0.43(±0.056) F09[N-O]
− 0.48(±0.043) CATS2D_05_AA + 0.44(±0.07)*SM14_AEA(dm)
− 0.495(±0.061) CATS2D_03_NL − 0.091(±0.036) RDF140v 
− 0.577(±0.097) CATS2D_07_AA grama + 1.255(±0.296) J_Dz(p) (1)

N_training_ = 147, R^2^ = 0.811, R^2^_adj_ = 0.800, Q^2^_LOO_ = 0.784, MAE = 0.402, r_m_^2^_LOO_ = 0.701, ∆r_m_^2^_LOO_ = 0.147, K_XX_ = 0.371, ΔK = 0.03. N_test_ = 37, R^2^_Pred_/Q^2^_(F1)_ = 0.792, Q^2^_(F2)_ = 0.769, Q^2^_(F3)_ = 0.763, RMSEP = 0.558, r_m_^2^_test_ = 0.685, ∆r_m_^2^_test_ = 0.167

**Figure 1:**
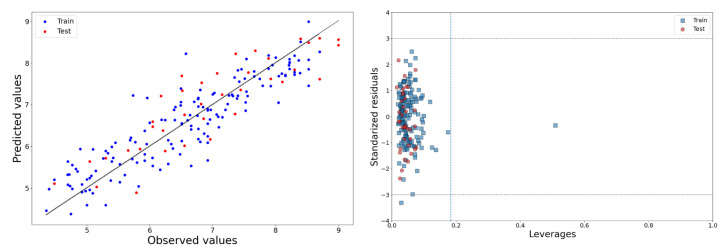
Observed vs. predicted activity (**left**) and Williams plot (**right**) of 2D-QSAR model.

The observed vs. predicted activity plot of the 2D-QSAR model is shown in Figure 1. However, as noticed by Gramatica et al. [25], it is also important to consider the difference between R^2^ and Q^2^_LOO_ in assessing internal predictivity. In this model, the R^2^ − Q^2^_LOO_ difference was found to be small (0.027), indicating good internal predictivity. Furthermore, the model achieved satisfactory values for the metrics r_m_^2^_LOO_ (= 0.701) and ∆r_m_^2^_LOO_ (=0.147), which are considered more stringent parameters than Q^2^_LOO_. For these parameters, acceptable values are greater than 0.50 and less than 0.20, respectively. Additionally, the low value obtained for MAE indicates that the model achieves sufficient internal predictivity.

Subsequently, to assess external predictivity, a test set of 37 compounds was used. The model demonstrated a relatively high value of 0.792 for predicting the biological activity of the test set compounds. The Q^2^_F2_ and r_m_^2^_test_ values further confirmed the model’s satisfactory performance on the test set. The low values obtained for RMSEP and ∆r_m_^2^_test_ also support the model’s external predictivity.

The maximum intercorrelation (R^2^) between any two descriptors in the model was found to be 0.557, indicating that the descriptors used in the model are independent. The intercorrelation matrix can be found in Table S4 of the Supplementary Materials. The variance inflation factor (*VIF*) was calculated for each descriptor, and none of the values exceeded 5.0, indicating the absence of multi-collinearity in the model. Moreover, the model exhibited acceptable K_xx_ and ΔK values, further supporting its robustness. The *Y*-randomization test with 1000 runs yielded a *cR_p_*^2^ value of 0.784, indicating that the model is not a result of chance but rather a unique and meaningful model.

The Williams plot of this 2D-QSAR model is presented in Figure 1. Only one training set compound was found to be a structural outlier and two compounds appeared as response outliers. Nonetheless, given the good predictivity of the structural outlier, we decided to retain it in the model.

**Figure 2:**
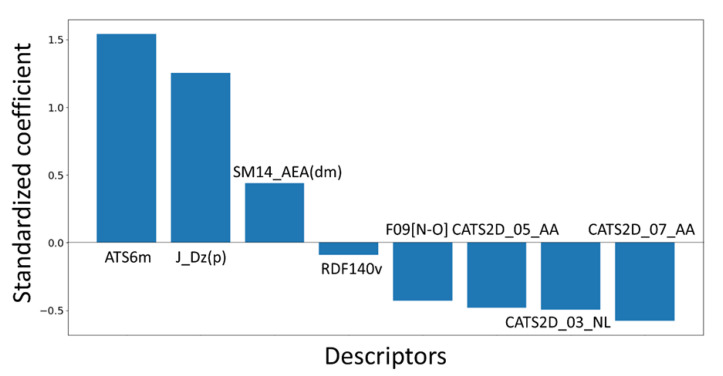
Relative significance of each descriptor of the 2D-QSAR model.

In Table 1, the eight descriptors of this 2D-QSAR model are listed along with their meaning, and their relative significance, determined by the standardized coefficients, is depicted in Figure 2. As can be noticed, save for RDF140v, all these descriptors belong to the category of 2D descriptors [26]. For example, ATS6m, J_Dz(p) and SM14_AEA(dm) are 2D topological descriptors, which provide information about the structural characteristics and connectivity patterns within the compounds.

Among the descriptors, ATS6m was identified as the most significant descriptor in the model, showing a positive correlation with pIC_50_(M). ATS6m is a 2D autocorrelation descriptor that encodes the distribution of atomic mass within a molecule, considering atom pair distances up to a 2D topological distance of 6 [27]. The analysis of the ATS6m descriptor values revealed that compounds with higher molecular weights and higher values of ATS6m tend to have a higher affinity towards the sEH enzyme. This indicates that both molecular weight and specific 2D topology, as encoded by the ATS6m descriptor, play important roles in determining the activity of the compounds as potent inhibitors of the enzyme.

The next most significant descriptor in the model is J_Dz(p), which is a 2D matrix-based descriptor representing the Balaban-like index from the Barysz matrix weighted by polarizability [26]. Similarly, a higher value of J_Dz(p) was found to be associated with higher biological activity. This suggests that, apart from molecular mass and topology, the polarizability of compounds may also play a crucial role in influencing their inhibitory activity against sEH.

**Figure 3:**
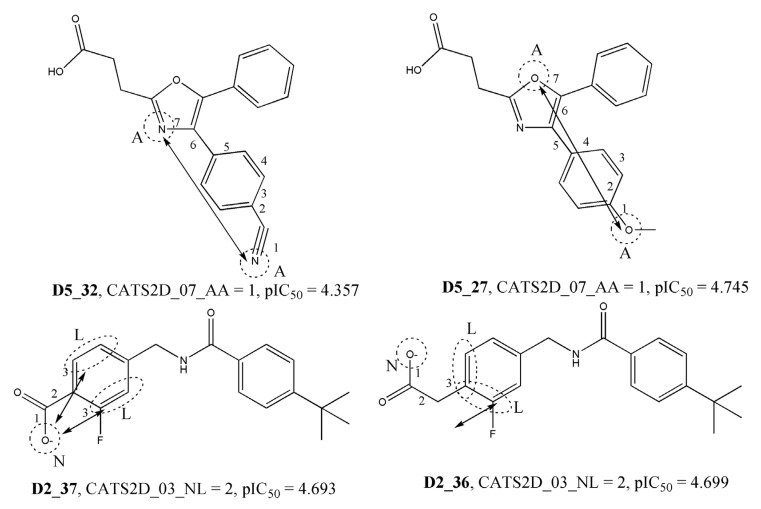
The negative influence of descriptors CATS2D_07_AA and CATS2D_03_NL for the biological activity of the compounds.

**Figure 4:**
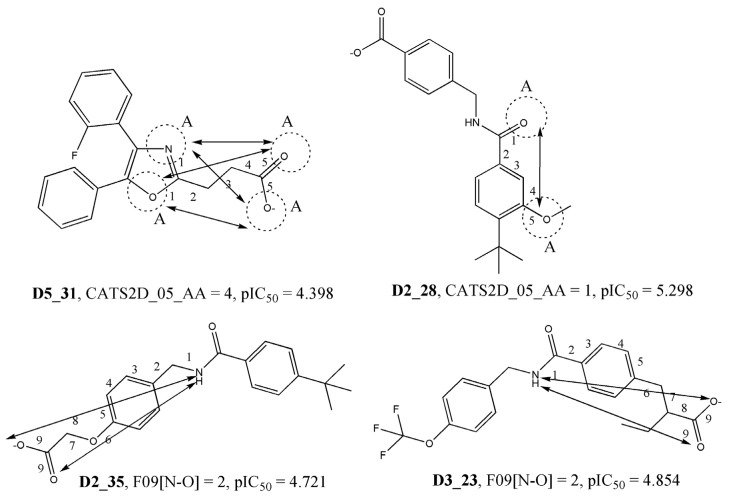
The negative influence of descriptors CATS2D_05_AA and F09[N-O] for the biological activity of the compounds.

The third, fourth, and fifth most significant descriptors of the model belong to the category of CATS2D or 2D pharmacophore descriptors, specifically CATS2D_07_AA, CATS2D_03_NL, and CATS2D_05_AA. Chemically advanced template search (CATS) descriptors are particularly useful in elucidating the structural requirements for higher activity. These descriptors encode the topological distances between specific pharmacophore features within the molecules [28]. For example, the descriptor CATS2D_07_AA indicates the presence of hydrogen bond acceptors (A) at a topological distance of 7. In the context of the 2D-QSAR model, this descriptor was found to negatively impact the endpoint response. This suggests that compounds with fewer hydrogen bond acceptors located at such a topological distance have higher biological activity. The following most significant descriptor of the model is F09[N-O]. This is a simple 2D atom-pair descriptor that specifically captures the frequency of nitrogen (N) and oxygen (O) atoms located at a topological distance of 9 within the compounds. It is interesting to note that, similar to CATS2D_07_AA, the F09[N-O] descriptor also exhibits a negative correlation with the endpoint response. This means that compounds with higher values of this descriptor tend to be less active, while most of the highly active compounds tend to have lower values of this descriptor. Indeed, as illustrated in Figure 3 and Figure 4, the observed correlation between higher descriptor values and lower activity for some compounds reinforces the importance of these descriptors in capturing the relevant structural features influencing the biological activity in the context of the 2D-QSAR model.

The last two descriptors, i.e., SM14_AEA(dm) and RDF140v, further contribute to the understanding of the compounds’ biological activity against sEH. SM14_AEA(dm) is a 2D graph-based descriptor weighted by the dipole moment (dm). The positive correlation between SM14_AEA(dm) and the biological activity suggests that compounds with higher dipole moments tend to exhibit higher inhibitory activity against sEH. This indicates that electrostatic interactions, mediated by the dipole moment, play a significant role in the binding of compounds to the target enzyme. On the other hand, RDF140v is a 3D descriptor (RDF) weighted by van der Waals volume (v). It captures the steric effects and the spatial distribution of atoms in the molecule. The negative correlation between RDF140v and the biological activity indicates that steric interactions, mainly governed by van der Waals volume, influence the binding and activity of compounds against sEH.

Interestingly, both J_Dz(p) and SM14_AEA(dm) exhibit a positive correlation with the biological activity, whereas, contrary to ATS6m, RDF140v shows a negative correlation. The contrasting correlations of these four descriptors (ATS6m, J_Dz(p), SM14_AEA(dm), and RDF140v) indicate the complex interplay of molecular topology, electrostatic interactions, and steric effects in shaping the biological activity of the compounds. Understanding these relationships can help in the design of compounds with optimized structural features to enhance their inhibitory activity against the target enzyme sEH.

In order to check whether non-linear models may be developed with better statistical predictivity, we attempted to develop some non-linear models using three distinct machine learning techniques, namely, MLP, RF, and SVM. A concise overview of the statistical outcomes derived from these models is shown in Table 2.

Clearly, none of the non-linear models managed to outperform the previously discussed linear 2D-QSAR model. Moreover, the non-linear models with the highest statistical significance were established using descriptors from the most predictive linear model (Equation (2)), through the utilization of MLP and SVM techniques. Remarkably, this SVM model was developed using a linear kernel. In contrast, descriptors chosen via differential Shannon entropy (dSe) proved insufficient to yield any models exhibiting statistical significance surpassing either the non-linear models or the proposed linear model.

### 2.2. Transformer-CNN-Based QSAR Model

The Transformer-CNN-based model yielded promising results in terms of its predictive performance and interpretability. The model attained a 5-fold cross-validated Q^2^ value of 0.713, coupled with an RMSE (CV) of 0.628, which underscore its ability to precisely forecast compound activity. This assertion is reinforced when the model is evaluated with a separate test set comprising 37 data points, yielding an R^2^_Pred_ of 0.731. This outcome thus further confirms its predictive power. This model was produced with 200 epochs and a batch size of 4. It is notable that increasing the batch size to 16, 32, or 64 compromised the predictivity of the model. Similarly, reducing the number of epochs to 100 or increasing it to 300 also resulted in reduced predictivity.

**Figure 5:**
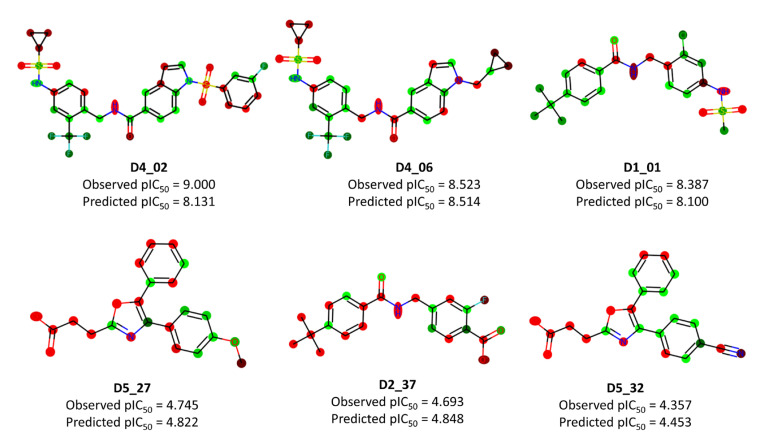
Structural interpretations obtained from the Transformer-CNN model for some selected highly active (upper row) and less active (lower row) dataset compounds. Color codes: green (favorable) and red (unfavorable).

In addition to predictive performance, the focus was also on the interpretability of the Transformer-CNN model. The LRP (Layer-wise Relevance Propagation) algorithm implemented in the Transformer-CNN repository was employed to obtain structural interpretations from the model. The interpretations for selected highly active and less active compounds from the dataset are depicted in Figure 5.

The insights extracted from the Transformer-CNN model offer valuable understandings into the structural characteristics influencing the activity against sEH. These interpretations align with the findings from the conventional 2D-QSAR model, which identified molecular mass and polarizability as important factors in governing higher activity. For example, compounds **D1_01** and **D2_37** are structurally similar, but the presence of a chlorine atom and sulfonamide (which contain heavy atoms and polar atoms) make a major difference to their activities. This observation is also consistent with the predictions of the conventional 2D-QSAR model, which highlighted the unfavorable effect of negative ionizable carboxylate for higher biological properties (see Figure 3). In the comparison of compounds **D5_27** and **D5_32**, despite their structural similarities, the contributions of oxazole atoms varied considerably. Similarly, the contributions of the methylbenzene scaffold also varied to a considerable extent in **D1_01** and **D2_37**. This reinforces the notion that it is the overall topology of the compounds that shapes their activity. The examples of compounds **D4_02** and **D4_06** also demonstrate the impact of specific structural features on activity. The contributions of the carboxamide group varied considerably between these compounds, indicating that this feature plays a significant role in their differential activities. The sulfonamide residues were generally found to be partially favorable, while only the oxygen atoms of the carboxamide contributed positively to higher biological properties. To corroborate these interpretations, we also contrasted the results of MD simulations for compounds **D4_02** and **D2_37** with the insights derived from the Transformer-CNN model. This comparison likely lends additional support to the relationship between identified structural features and their impact on the compounds’ activity against sEH.

The color codes depicted in Figure 5 hold varying significance based on the LRP algorithm. For compounds **D1_01** and **D2_37**, which are structurally akin, the relevance of each atom concerning favorable and unfavorable activity is shown in the Supplementary Materials. It is evident that **D2_37** exhibits maximum negative influence primarily from its fluorine atom and the oxygen atom of the carboxylate. On the contrary, the chlorine atom in **D1_01** contributes significantly and positively to its higher potency. Notably, the negative influence of the carboxamide fragment is markedly more pronounced in **D1_01** than in **D2_37**.

### 2.3. 3D-QSAR Analysis

**Figure 6:**
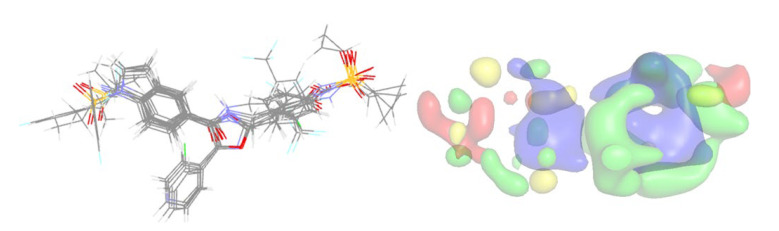
The aligned structures of the compounds used for 3D-QSAR modeling (**left**) and all contour maps (**right**). Notice that only the five best actives and the five least actives are shown. Color codes: green (steric favorable), yellow (steric unfavorable), blue (electropositive favorable), and red (electronegative favorable).

The current 2D-QSAR model illustrates the significance of steric and electrostatic interactions, as well as specific fragments and pharmacophores, in determining the activity against sEH. To gain a better understanding of the structural requirements, we resorted to 3D-QSAR modeling and analysis using the Open3DQSAR software. Similar to the 2D-QSAR modeling approach, the dataset was randomly divided into a training set and a test set. Atom-based rigid body alignment was performed to align the structures, which were then used to calculate steric and electrostatic fields. Two different feature selection techniques, FFD-SEL and UVE-PLS, were employed for PLS model development. Both techniques yielded the most predictive models with three components. Figure 6 presents the aligned structures and contour maps, while Table 3 showcases the statistical results of the models.

The UVE-PLS technique yielded superior statistical results in the 3D-QSAR analysis conducted with a training set of 148 compounds and a test set of 36 compounds. The model achieved satisfactory Q^2^_LOO_ (=0.643) and R^2^_Pred_ (=0.657), considering the inclusion of a relatively large and structurally diverse dataset, potentially involving multiple binding mechanisms. The UVE-PLS model indicated that electrostatic interactions (34%) and steric contributions (66%) played a significant role in determining the binding affinity of the ligands towards sEH, with the steric component being dominant. Unlike 2D-QSAR models, assessing the applicability domain of 3D-QSAR models is challenging. However, leverage values of the training set compounds were determined using the Open3DQSAR tool, and it was observed that the leverage values (range: 0.983–0.849) did not vary considerably. Hence, it can be assumed that the compounds analyzed in this study were well within the AD of the model.

**Figure 7:**
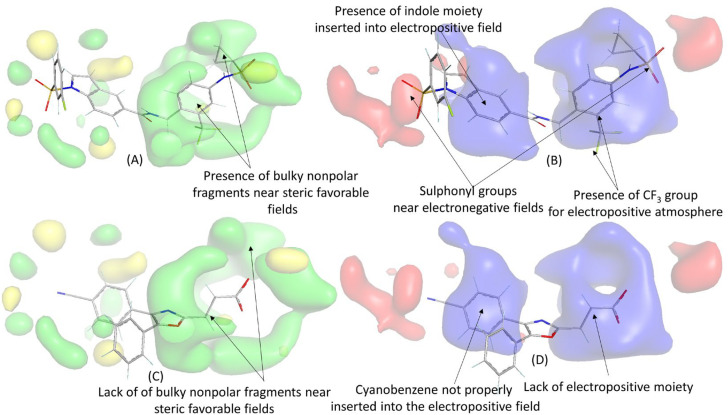
3D-QSAR contour maps for one of the most potent compounds (**D4_02**) and the least potent compound of the dataset (**D5_32**): (**A**) electrostatic maps of **D4_02**, (**B**) electrostatic maps of **D4_02**, (**C**) steric maps of **D5_32**, (**D**) electrostatic maps of **D5_32**. Color code: green (steric favorable), yellow (steric unfavorable), blue (electropositive favorable), and red (electronegative favorable).

**Figure 8:**
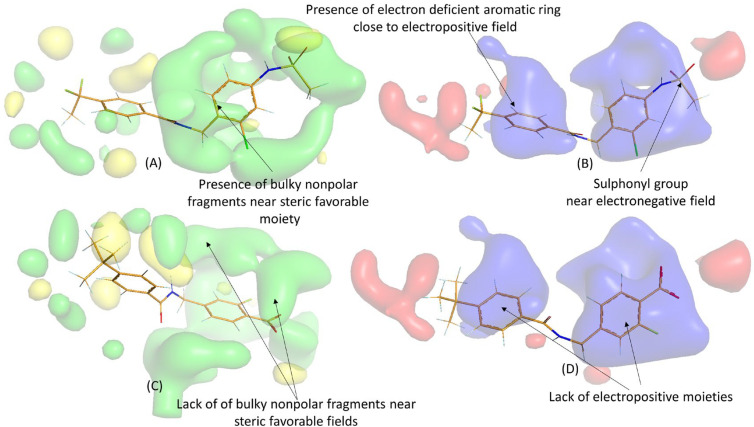
3D-QSAR contour maps for one of the most potent compounds (**D1_24**) and one of the least potent compounds of the dataset (**D2_37**): (**A**) electrostatic maps of **D1_24**, (**B**) electrostatic maps of **D2_37**, (**C**) steric maps of **D1_24**, (**D**) electrostatic maps of **D2_37**. Color code: green (steric favorable), yellow (steric unfavorable), blue (electropositive favorable), and red (electronegative favorable).

Figure 7 displays the most potent compound (**D4_02**) and the least potent compound (**D5_32**) from the dataset, along with their respective contour maps. An analysis revealed that the bulky aromatic moiety of **D4_02** is positioned near the steric favorable field, whereas such bulky groups are absent in **D5_32.** This indicates that steric interactions play a significant role in the potency of **D4_02**, which is consistent with our findings in the 2D-QSAR models, where descriptors such as ATS6m and RDF140v emerged as important factors. Additionally, electropositive (electron-deficient) fields were more prevalent than electronegative (electron-rich) fields. In the case of **D4_02**, the presence of the trifluoromethyl group in the benzene ring created an electropositive environment, which was absent in **D5_32**. Furthermore, **D4_02** featured an indole ring fully inserted into another electropositive field, whereas the cyanobenzene residue of **D5_32** (with an electron-deficient benzene residue) was not fully inserted into this field. Please note that this information may not be visible in Figure 8, and an additional figure from a different angle is provided in the Supplementary Materials (Figure S3).

It is evident that, in addition to the presence of polar groups, the specific topology of the compounds plays a crucial role in governing their biological activity, as also suggested by the 2D-QSAR analysis. Interestingly, two sulphonyl residues of **D4_02** were found to be in proximity to the electronegative favorable contour maps, which could further enhance the biological activity of this molecule. Conversely, no electron-rich group was observed near these contours. Similar observations were made when examining the contour maps of higher active compounds **D1_24** and **D2_37**, as depicted in Figure 8.

### 2.4. Molecular Dynamics Simulations

The compounds **D4_02** and **D2_37**, which represent one of the most potent and one of the least potent compounds, respectively, were subjected to 50 ns molecular dynamics (MD) simulations. These compounds were docked into the active site of the sEH protein (PDB: 4X6X). Similarly, the complex 4X6X with a bound ligand (**S74**: 3-{4-[(1-{[(1s,2R,3S)-2,3-diphenylcyclopropyl]carbamoyl}-piperidin-4-yl)oxy]phenyl}-pro-panoic acid) was used as a reference protein complex for MD simulations. However, prior to conducting molecular docking on the dataset compounds, a self-docking analysis was performed using **S74** in the 4X6X configuration. This step aimed to validate the docking methodology, resulting in an RMSD of 1.54 Å between the docked pose of **S74** and its bound pose.

**Figure 9:**
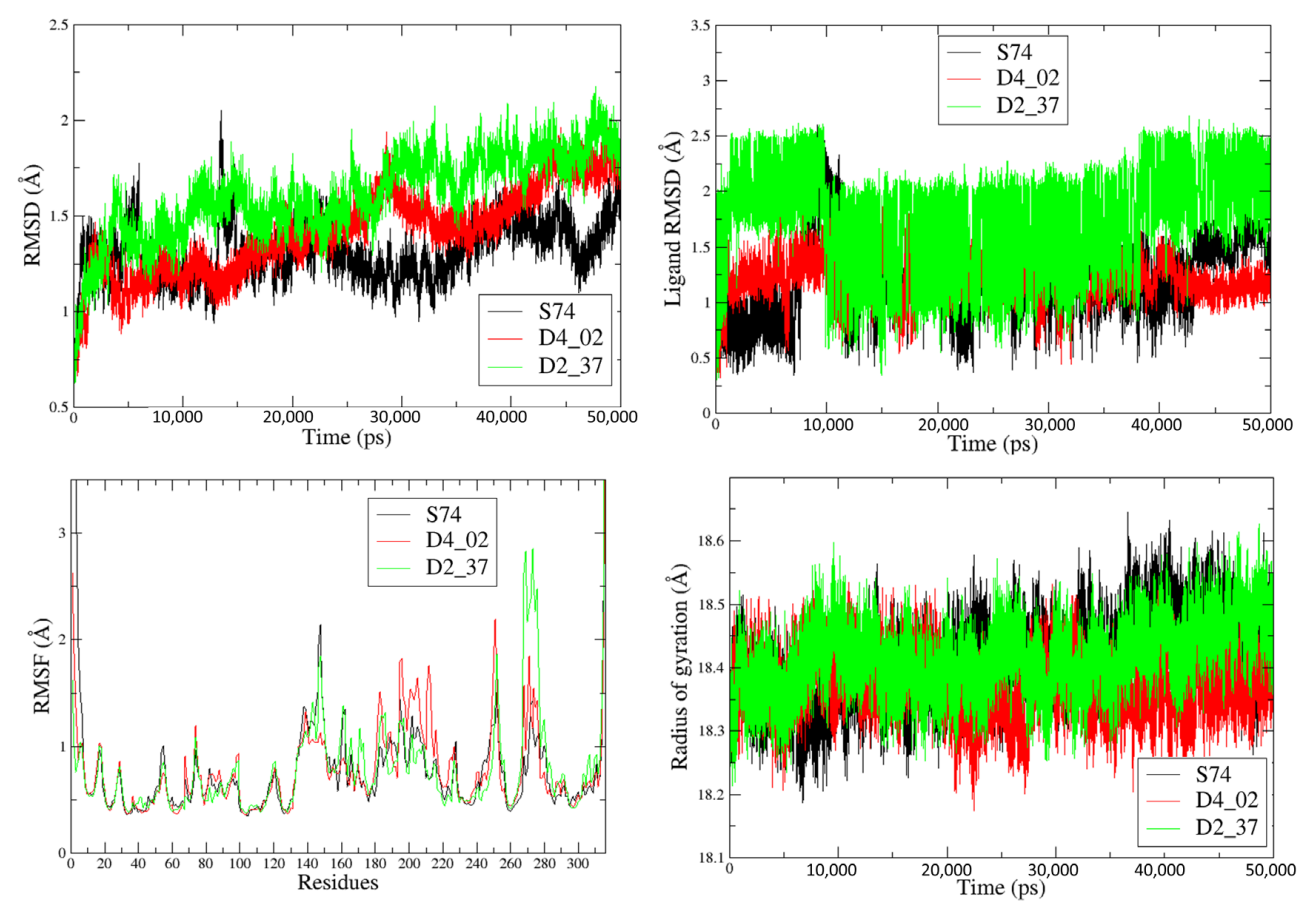
Results from the trajectory analysis for the MD simulations of **D4_02** and **D2_37** as well as of **S74** (bound ligand of PDB 4X6X).

Figure 9 shows the RMSD plots of the protein backbones and ligands, along with the RMSF and RG plots. From the ligand RMSD plots, it is apparent that the highly active compound **D4_02** exhibits lower fluctuations compared to the less active compound **D2_37**, primarily due to the lower fluctuations of **D4_02** in residues 140–160 and 260–280. However, **D4_02** displays higher fluctuations in residues 180–220 when compared to both **S74** and **D2_37**. When assessing the compactness of the complexes using the radius of gyration (RG) plots, it was observed that the **D4_02**-4X6X complex remained more compact throughout the MD simulation compared to the **D2_37**-4X6X complex.

We also calculated the MM-GBSA binding energies for these complexes, which are presented in Table 4. The results clearly indicate that the highly active compound **D4_02** exhibits a higher binding affinity towards sEH compared to the less active compound **D2_37**, consistent with the ligand RMSD plots of these two compounds. Compound **D4_02** showed higher electrostatic and van der Waals interactions, and notably, there were significant differences in electrostatic interactions (ΔE_elec_) between **D4_02** and **D2_37**. This finding aligns with our 3D-QSAR analyses, which suggested that electrostatic interactions play a substantial role in determining the inhibitory potentials of the compounds in the dataset. The lower entropy of **D4_02** contributed to its higher theoretical binding energy. Given these findings, it was essential to examine the final binding poses obtained for these two compounds in the analysis.

**Figure 10:**
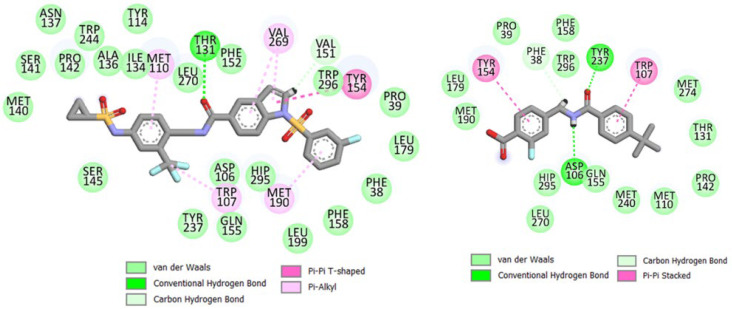
Poses obtained from the final trajectory of MD simulations for **D4_02** and **D2_37**.

Figure 10 displays the final binding poses of compounds **D4_02** and **D2_37**. The 3D-QSAR analysis correctly predicted the involvement of π-π and π-alkyl interactions between the indole moiety of **D4_02** and amino acid residues such as Tyr154, as well as Val269 (due to its insertion into an electropositive favorable field). Similarly, the π-alkyl interactions of the trifluoromethylbenzene moiety were well predicted by the 3D-QSAR model. While both aromatic rings of **D2_37** exhibited π-π interactions with the amino acid residues, the overall van der Waals and electrostatic interactions of this ligand were significantly lower than those of **D4_02**. It should be noted that our 3D-QSAR analysis accurately predicted a large number of van der Waals interactions surrounding the trifluoromethylbenzene moiety of **D4_02** (cf. the steric favorable field). In contrast, fewer van der Waals interactions were observed in **D2_37** due to its lower molecular mass, which was also indicated by the 2D-QSAR model where ATS6m was identified as the most influential descriptor.

Furthermore, **D4_02** exhibited hydrogen bond interactions with Thr131, whereas **D2_37** depicted hydrogen bond interactions with Tyr237 and Asp106. It is worth noting that these interactions were not predicted by the 3D-QSAR model, likely because most of the compounds in the dataset had amide moieties that were aligned, and these specific interactions were not found to have a significant influence in the 3D-QSAR analysis.

Finally, it is important to compare the interpretation results from the Transformer-CNN with the interactions obtained from the MD simulations. The Transformer-CNN accurately predicted the interactions of the carboxamide, trifluoromethyl, and aromatic rings. Notably, the carboxylate group of **D2_37** was solvent-exposed and did not show polar interactions with amino acid residues. This lack of polar interactions contributed to the unfavorable ΔE_elec_ of this compound, thereby reducing its overall binding affinity. This observation may explain the negative influence of carboxylate residues in both the 2D-QSAR and Transformer-CNN models. Additionally, one of the sulphonyl groups of **D4_02** formed a hydrogen bond interaction with Gln155 (not shown in Discovery Studio Visualizer but detected by the PoseView software of https://proteins.plus/, (accessed on 3 June 2023) and presented in Figure S2 of Supplementary Materials).

## 3. Materials and Methods

### 3.1. Conventional 2D-QSAR Modeling

#### 3.1.1. Dataset Collection and Preparation

The dataset utilized in this study consists of 184 structurally diverse human soluble epoxide hydrolase (sEH) inhibitors, which were sourced from articles published by research groups affiliated with the Goethe University, Germany [4,10,14,16,29]. A complete listing of the SMILES of the dataset compounds, along with the corresponding experimental data, can be found in Table S1 of the Supplementary Materials. The chemical structures of the inhibitors were obtained either from the provided SMILES notations in the original publications or drawn using ChemSketch [30]. These canonical SMILES were subsequently converted to .sdf format and protonated at pH 7.4 using the Openbabel software-2.4.1 [31]. To ensure consistency, the structures were converted back to canonical SMILES notation using the sdftosmi.py program from the tanimoto_similarities package (https://github.com/MunibaFaiza/tanimoto_similarities, accessed on 10 June 2023), and any duplicate structures were removed. Further processing of the .sdf structures was performed using Chemaxon in the OCHEM platform, involving the following steps: (a) standardization, (b) neutralization, (c) removal of salts, and (d) cleaning of structures [32]. Furthermore, geometrical optimization of the structures for the calculation of 3D descriptors was conducted using Corina under the OCHEM platform [33].

To assess the structural diversity of the dataset compounds, we generated their MACCS Keys structural fingerprints [34]. These fingerprints were employed to compute a distance matrix using Tanimoto Similarity analysis. Subsequently, the distance matrix underwent t-Distributed Stochastic Neighbor Embedding (t-SNE) analysis, producing a structural diversity plot with two components [35]. Following this, a k-means cluster analysis was performed using 6 clusters determined by the Silhouette score, resulting in a plotted representation (refer to Figure S1). Such representation clearly depicts that these structures cover a considerably large chemical space that can easily be clustered. 

The biological activity of interest here is the measured inhibitory potential of the compounds against human sEH, expressed as IC_50_ (in µM). The latter, as is usual, was log-converted (pIC_50_ (M) = −log_10_(IC_50_/10^6^)) and taken as the response variable for practical use in the subsequent 2D-QSAR modeling. 

#### 3.1.2. Calculation of Descriptors

Various descriptor-calculating tools were employed in this study to calculate the descriptors for the compounds. These tools include: (a) AlvaDesc v.2.0.4 [36]; (b) CDK 2.7.1 [37]; (c) GSFragments plus ISIDA fragments [38]; (d) MORDRED [39]; (e) Multilevel Neighborhoods of Atoms (MNA) [40]; (f) Simplex representation of molecular structure—SIRMS (https://github.com/DrrDom/sirms, accessed on 10 April 2023); (g) MERA + MERSY [41]; (h) RDKit (https://www.rdkit.org/, accessed on 10 April 2023); and (i) PyDescriptors [42].

All these descriptors were calculated using the OCHEM web platform [32]. Each set of descriptors was employed separately to develop QSAR linear interpretable models. These models will be specifically referred to as 2D-QSAR models to distinguish them from the other QSAR modeling approach applied in this study.

#### 3.1.3. Dataset Division and Feature Selection

The dataset was divided into a training set and a test set using the open-access Python-based SFS-QSAR tool (available at https://github.com/ncordeirfcup/SFS-QSAR-tool_v2, accessed on 11 April 2023) [43]. The SFS-QSAR tool implements the *train_test_split* function from Scikit-learn [44], and a seed value of 3 was set to ensure reproducibility for each descriptor set. Two distinct feature selection techniques were employed to generate the linear 2D-QSAR models by adopting a multiple linear regression (MLR)-based procedure, namely: (i) Sequential Forward Selection (SFS) [43], and (ii) Genetic Algorithm (GA) [45].

**Figure 11:**
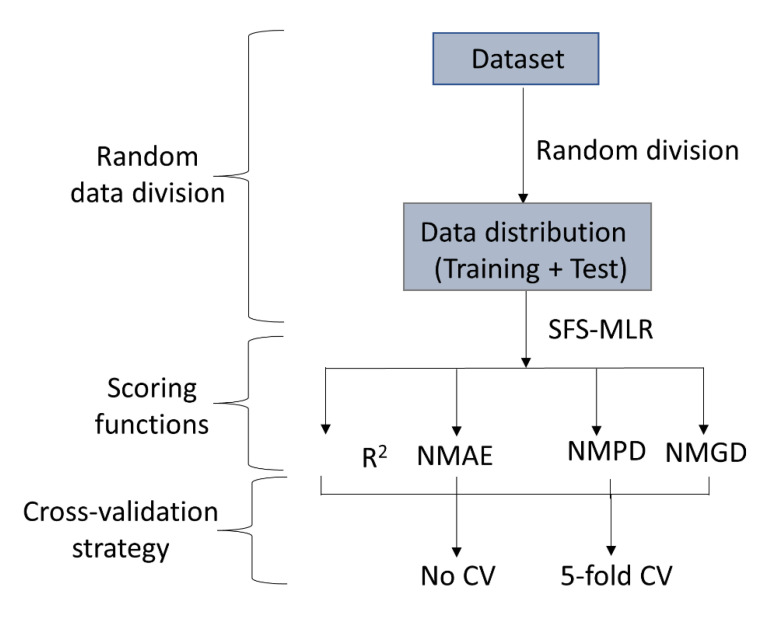
Model development strategy adopted for developing SFS-MLR models.

Feature selection is an important step in developing linear QSAR models as it identifies the most significant descriptors for determining the structural requirements of the compounds. SFS is a non-stochastic feature selection method that consistently produces the same model given the same descriptors, data distribution, and parameter settings. In this study, the SFS-MLR models were developed using the open-access SFS-QSAR-tool, which implements the Mlxtend tool (http://rasbt.github.io/mlxtend/, accessed on 5 April 2023). Four scoring functions, i.e., determination coefficient (R^2^), negative mean absolute error (NMAE), negative mean Poisson deviance (NMPD), and negative mean gamma deviance (NMGD), were chosen one by one in this tool, with the option of no cross-validation (No CV) or 5-fold cross-validation (5-fold CV). As a result, eight SFS-MLR models were generated for each descriptor set, as shown in Figure 11.

Conversely, GA is a stochastic method. The GA-MLR models were created using the GeneticAlgorithm v.4.1_2 open-access tool [45] with default settings, including 100 iterations/generation, a crossover probability of 1, a mutation probability of 0.3, an initial number of 100 generated equations, and the selection of 30 equations in each generation. GA involves the random selection of descriptors, estimation of fitting scores for these random models, and the application of crossover and mutation schemes to improve the fitting scores and establish the final models [45]. To account for the stochastic nature of GA, at least 20 different runs were performed for each dataset, and the best model was selected based on its overall predictivity.

Before model development, a pre-treatment step was performed in both tools. This involved setting a correlation cutoff of 0.99 and a variance cutoff of 0.0001 to eliminate highly correlated descriptors and constant/near-constant descriptors. For all linear interpretable 2D-QSAR models, a maximum of eight descriptors was allowed.

#### 3.1.4. Model Evaluation

In order to compare the statistical quality of the developed models and determine the most reliable one, two well-known validation parameters were utilized, namely Q^2^_LOO_ (leave-one-out cross-validated determination coefficient R^2^) [23] and R^2^_Pred_ (predicted R^2^ or Q^2^_F1_) [24]. The former is known for evaluating the internal predictivity of the model, whereas the latter estimates its external predictivity. The average value of these parameters was considered to select the most statistically reliable model.

To further assess the final models, additional statistical parameters were employed, i.e., the adjusted R^2^ (R^2^_Adj_), the Fisher statistic (F-test), the mean absolute error (MAE), and the metrics rm^2^_LOO_ and ∆rm^2^_LOO_ were computed for the training set, whereas Q^2^_(F2)_, Q^2^_(F3)_, the root mean square error of prediction (RMSEP), and the metrics rm2test and ∆rm2test were computed for the test set. These parameters provide a more critical evaluation of the final models, both in terms of internal performance and external predictivity. A detailed description of these statistical parameters can be found elsewhere [25,46,47].

Likewise, to ensure the robustness and reliability of the proposed 2D-QSAR models, additional tests were carried out. To begin with, the maximum inter-collinearity among the descriptors of the final models was estimated from the cross-correlation matrix using the SFS-QSAR-tool. Then, the multi-collinearity of the final models was assessed using the variance inflation factor (*VIF*) [48], defined as follows:

(2) $$VIF=\frac{1}{\left(1-R_{i}^{2}\right)}$$

where $R_{i}^{2}$ is the coefficient of determination (*R*^2^) obtained from regressing the *i*^th^ descriptor on the other descriptors [48].

The multi-collinearity of the 2D-QSAR models was also checked using the parameters K_xx_ and ΔK calculated by the software QSARINS v2.2.4 [49]. The K_xx_ parameter represents the overall correlation among descriptors, while ΔK is the difference between the correlation among descriptors (K_x_) and the correlation between descriptors and responses (K_xy_) [50].

To further ensure the statistical robustness of the models, a *Y*-randomization test was performed. This involved randomizing the response variables while keeping the descriptors unchanged and then calculating the *cR_p_^2^* value using the following formula [51]:

*cR_p_^2^* = R√ (R^2^ − R_r_^2^) (3)

where R_r_ denotes the average R^2^ obtained from the randomized models. A value of cR_p_^2^ greater than 0.5 generally suggests that the model was not developed by chance [51].

#### 3.1.5. Applicability Domain of the Models

The applicability domain of a QSAR model refers to the region in the response and chemical structure space in which the model can make reliable predictions for new or unseen compounds [46]. In this work, to determine the applicability domain (AD) of the 2D-QSAR models, a leverage estimation approach was followed, and the Williams plot generated. The Williams plot displays the leverage, which measures the influence of individual data points, against the standardized residuals [46,52,53,54]. This plot helps in identifying structural and response outliers in the linear 2D-QSAR models. It is important to note that, according to the Organization for Economic Cooperation and Development (OECD) guidelines, QSAR models should be reported along with their applicability domain. This ensures that the reliability and validity of the models can be assessed based on their performance within the defined applicability domain [22].

#### 3.1.6. Machine Learning Techniques and Partial Least Square (PLS)

Non-linear models were set up using selected features via three distinct techniques: (a) multilayer perception (MLP) [55], (b) support vector machines (SVM) [56], and (c) random forests (RFs) [57]. These models were developed using the open-source software “Non-linear-Regression-tools” (available at https://github.com/ncordeirfcup/Non-linear-Regression-tools, accessed on 15 May 2023), which leverages Scikit-learn-based programs for model creation while incorporating hyperparameter optimization. Within this tool, users can specify the necessary parameters by means of a .csv file. These parameters are then tuned to create optimal models based on 5-fold cross-validation on the training set. The optimized parameters for this study are detailed in Table S2 of the Supplementary Materials. The performance of the final models is subsequently gauged against external predictivity with the test set. Two distinct feature selection algorithms were employed during the development of the non-linear models. Firstly, descriptors from the most predictive linear model were utilized for setting up the model. As an alternative, we identified the eight most significant descriptors using differential Shannon entropy, a process implemented through the open-access tool IMMAN [58].

Additionally, the partial least squares (PLS) method was also employed using the selected features. This procedure was facilitated by another open-access tool named PLS-QSAR (accessible at https://github.com/ncordeirfcup/PLS-QSAR, accessed on 15 May 2023), resourcing to the following settings: maximum number of components: 5, condition: “CVLOO” (cross-validation leave-one-out), and increment: 5. Therefore, the tool would cease further component addition if the inclusion of an extra component fails to improve the Q^2^_LOO_ value for the training set by at least 5%.

#### 3.1.7. Consensus Modeling

We utilized the “Intelligent Consensus Prediction” (ICP) technique where multiple predictive models are coupled to check if their combinations improve the external predictivity. A more in-depth description of the ICP methodology applied in the current investigation can be found elsewhere [59,60]. In summary, this technique encompasses four consensus prediction approaches: (a) CM0: an ordinary consensus formed by calculating the arithmetic average of predicted values from all individual models; (b) CM1: the average predictions derived from all qualified models; (c) CM2: weighted average predictions computed from all qualified models; and (d) compound-wise best selection of predictions from individual models. All consensus models were generated using the open-access Java-based “Intelligent Consensus Predictor” tool, available at https://sites.google.com/site/dtclabicp/, (accessed on 20 July 2023).

### 3.2. Transformer-CNN Based QSAR Modeling

Transformer-CNN (Convolutional Neural Network) is a powerful machine learning architecture for QSAR modeling and interpretation recently introduced by Karpov et al. (available at https://github.com/bigchem/transformer-cnn, accessed on 22 May 2023) [61]. A detailed and in-depth description of its methodology and related code can be found in the author’s original work. Briefly, the dataset consists of compounds represented as SMILES strings, and the process begins with SMILES-embedding following an encoder approach, similar to a machine translation problem. To do so, a convolution neural network is employed to perform SMILES canonicalization in a Sequence-to-Sequence (Seq2Seq) manner, in which the left side consists of non-canonical SMILES and the right side includes their corresponding canonical counterparts. After encoding, the extracted latent variables effectively represent relevant features that can be applied to QSAR modeling.

Specifically, the SMILES strings are transformed into dynamic SMILES embedding of size 64, with variable lengths, which are then subjected to 1D convolutional filters. The convolutional filters have different kernel sizes ranging from 1 to 20, with corresponding numbers of filters assigned to each size [61]. Next, a global max-pooling operation is performed, and the pooling results are concatenated. The data then go through Dropout (rate = 0.1), Dense (N = 512 neurons and using the activation function “Relu”), and Highway NN layers (N = 512 neurons and using the activation function “Sigmoid”), before reaching the output layer. Typically, the weights of the Transformer’s part are kept frozen, and the regression models are built by applying the Adam optimizer and checking the Mean Squared Error (MSE).

In this work, following the authors’ suggestions [61], 10 non-canonical SMILES were generated for each data-point using RDKit (https://www.rdkit.org, accessed on 20 May 2023), which were processed by one-hot encoding (using 66 symbols) for setting up the Seq2Seq inputs. The training of the Transformer-CNN model involved variable learning rates for a specified number of epochs (*n* = 100) and a batch size of 4. Early stopping was applied using 10% randomly selected SMILES to identify the optimal model. To mitigate overfitting, cross-validation techniques were employed.

While Transformer-CNN is available on the OCHEM web platform, in this work, the models were built using the Python-based tool provided on the GitHub repository. However, the tool codes were slightly modified to accommodate the upgraded version of TensorFlow (https://www.tensorflow.org/, accessed on 22 May 2023). The model was initially built using the same training set as that used while developing the final 2D-QSAR model, with the help of the transformer-cnn.py script. A configuration file (*config.cfg*) was used to specify input data, canonization option, seed value, number of epochs, batch size, and output data file name. A 5-fold cross-validation of the generated model was performed using the *cv5.sh* bash script with the configuration file (*config-cv.cfg*). Finally, the test dataset was employed to estimate the external predictivity of the generated models using a different configuration file (*config_val.cfg*). The input configuration files for these steps are provided in the Supplementary Information for reproducibility.

To interpret the models and assess the significance of individual input features, the “standalone” Transformer-CNN tool was applied (available at https://github.com/bigchem/transformer-cnn, accessed on 22 May 2023). This tool utilizes the Layer-wise Relevance Propagation (LRP) algorithm, which splits the overall predicted result into a sum of contributions coming from the individual neurons. The relevance is propagated from the last layer to the input layer, allowing the evaluation of contributions from specific input variables and the identification of significant features for the training set or the explanation of individual neural network predictions [61].

### 3.3. 3D-QSAR Modeling

#### 3.3.1. Alignment Techniques

For the development of the 3D-QSAR models, the compounds in the dataset were aligned using an atom-based alignment method or unsupervised rigid body molecular alignment. Initially, the 3D structures of the ligands in the dataset were minimized using the “obminimize” function of OpenBabel. The minimization process involved employing the steepest descent technique and the MMFF94 forcefield [31]. After the minimization, the ligand structures were used to generate 100 conformations using the *rdMolAlign.GetCrippenO3A* code of Rdkit. The Python script “alignment.py” written and used for the atom-based alignment can be found in the GitHub repository: https://github.com/ncordeirfcup/InsilicoModeling_RdRp, (accessed on 25 May 2023) [52].

#### 3.3.2. Model Development

The 3D-QSAR models were generated using the aligned conformations with the open-source software called Open3DQSAR-2.24. The methodology for this software has been described in detail in earlier works by Tosco and Balle [62,63]. Open3DQSAR utilizes a carbon and a volume-less positively charged probe to estimate steric and electrostatic domains, respectively. In its data pre-treatment stage, a smart region definition (SRD) cut-off level (here equal to 2.0) is employed, and N-level variables are removed. Open3DQSAR deploys SRD for grouping variables. Two different variable selection algorithms are utilized for such a purpose, namely, Fractional Factorial Design-based variable SELection (FFD-SEL), and Uninformative Variable Elimination-based Partial Least Square (UVE-PLS).

To evaluate the predictive performance of the 3D-QSAR-oriented PLS models, several metrics were used, including the determination coefficient (R^2^), F-test result, leave-one-out Q^2^ (Q^2^_LOO_), leave-two-out Q^2^ (Q^2^_LTO_), leave-many-out Q^2^ (Q^2^_LMO_ with 5 groups and 20 runs), and, finally, R^2^_Pred_. The contour maps were examined using isocontour values at PLS coefficients of +0.002 (green) and −0.002 (yellow) for steric fields, and +0.001 (blue) and −0.001 (red) for electrostatic fields.

#### 3.3.3. Molecular Docking and Molecular Dynamics Simulations

The X-ray crystal structure of sEH hydrolase (PDB: 4X6X) [64] was downloaded and utilized for molecular docking of the selected compounds from the dataset. The docking was performed using the AutoDock 4.2 package [65]. A grid box with a spacing of 0.375 and dimensions of 50 Å × 50 Å × 50 Å was defined at the coordinates X = 2.98, Y = 4.39, and Z = 35.20. The detailed methodology for the docking procedure can be found in our previous work [66].

The best poses of the compounds obtained from the docking experiment were selected, and the resulting ligand–receptor complexes underwent 50 ns molecular dynamics (MD) simulations using Amber 20. The specific steps of the MD simulations were described in detail in our previous investigations [66,67]. Trajectory analysis was performed using the *cpptraj* function of Python. Various properties, such as the root mean square deviation (RMSD) of the complexes and ligands, the root mean square fluctuations (RMSF), and their radius of gyration (Rg), were calculated. To estimate the binding free energies of the complexes, the molecular mechanics generalized born surface area (MM-GBSA) approach was applied. The MM-PBSA.py tool was used to calculate the binding free energies, considering 100 snapshots taken from the last 10 ns of the MD production run. Additionally, the entropy contributions (−TΔS) to the binding free energies were determined using normal mode analysis, collecting 100 snapshots from the last 10 ns [67,68].

## 4. Conclusions

The sEH (soluble epoxide hydrolase) enzyme is indeed a significant biological target for various diseases. It has been identified that the binding affinity of sEH inhibitors can be influenced by the binding sites present in the enzyme’s C-terminal region, responsible for hydrolase activity, as well as the N-terminal region, associated with phosphatase activity. These distinct binding sites offer potential opportunities for designing and developing sEH inhibitors as single-target or multi-target agents, aiming to modulate the enzyme’s activity and provide therapeutic benefits in the context of different diseases. The understanding of these binding sites and their contributions to the inhibitory potency of compounds is crucial for the rational design of effective sEH inhibitors.

In the present work, a large and diverse series of sEH inhibitors were investigated using receptor-independent 2D-QSAR and 3D-QSAR analyses. The aim was to generate validated and predictive models that can provide insights into the structural requirements of these inhibitors. The most predictive linear 2D-QSAR regression model found achieved high predictive power, explaining 80% of the variances in the training set compounds and predicting 78.4% of their variances. More importantly, external validation on the test set compounds yielded a prediction of 79.4% for their variances. The model highlighted the importance of 2D pharmacophoric information, as indicated by its CATS2D descriptors, and emphasized the significance of topological characteristics and properties such as molecular mass, van der Waals volume, dipole moment, and polarizability in determining the biological activity against sEH. The transformer CNN-based model provided a clear pictorial understanding of favorable and unfavorable fragments responsible for biological potency. The 3D-QSAR analyses also demonstrated satisfactory statistical predictivity and supported the interpretations from the 2D-QSAR models. These analyses provided valuable information about the structural requirements of potent sEH inhibitors. Furthermore, the MD simulations conducted with highly active and less active compounds revealed important receptor–ligand interactions, which were consistent with the predictions from the QSAR models. This comprehensive investigation serves as an important guideline for the design of novel sEH inhibitors. For instance, the generated 2D-QSAR models can serve as a means to obtain average predicted values for novel compounds to be synthesized. To set up the 2D-QSAR linear models, along with generating plots and values, one can refer to the files “2DQSAR_train.csv” and “2DQSAR_test.csv” (accessible at https://github.com/amitporto/soluble-epoxide-hydrolase-inhibitors, accessed 2 August 2023) and process them either through the Flask-based web application accessible at https://amit-mlr.onrender.com, (accessed 2 August 2023) (note that when using this application, file names should remain unchanged) or simply by employing the SFS-MLR-tool, which is available at https://github.com/ncordeirfcup/SFS-QSAR-tool_v2, (last accessed on 5 August 2023). Both these tools are suitable for predicting outcomes for new compounds. In addition, the Transformer-CNN can be leveraged for predictions using the “sEH.pickle” object file (located at https://github.com/amitporto/soluble-epoxide-hydrolase-inhibitors, accessed on 2 August 2023) and the “ochem.py” script (accessible at https://github.com/bigchem/transformer-cnn/tree/master/standalone, accessed on 22 May 2023). Concerning the 3D-QSAR models, these offer a means to further predict activity as well as to check the proximity of molecular scaffolds along with favorable contour maps (steric and electrostatic contours can be found in files “uvepls_coefficients_fld-01_y-01.grd” and “uve-pls_coefficients_fld-02_y-01.grd” at https://github.com/amitporto/soluble-epoxide-hydrolase-inhibitors, accessed on 2 August 2023). Regarding the results of the MD simulations, these clearly demonstrated reduced fluctuations in amino acid residues 260–280 for both active complexes, pertaining to the dataset compound **D4_02** and bound ligand **S74**. This observation implies that stronger interactions with these amino acid residues could contribute to enhanced inhibitory potential. Substantial differences were observed in steric and electrostatic interaction energies between **D04_02** and **D2_37**. These factors should be monitored preliminarily while predicting the potency of the new compounds against the sEH enzyme.

Finally, and of paramount significance, the entirety of this study was undertaken using non-commercial open-access tools and web platforms to ensure fast reproducibility and accessibility. In the updated version of the SFS-QSAR-tool (available at https://github.com/ncordeirfcup/SFS-QSAR-tool_v2, accessed on 5 August 2023), we have incorporated two Jupyter notebook files, specifically, *multiSFSQSAR_random.ipynb* and *multiSFSQSAR_random.ipynb*. These notebooks were designed to assist users in generating multiple SFS-MLR models in a single run, as we performed in this study. Furthermore, we report here, for the first time, two automated Python-based tools, namely, Non-linear-Regression-tools (accessible at https://github.com/ncordeirfcup/Non-linear-Regression-tools, accessed on 15 May 2023) and PLS-QSAR (available at https://github.com/ncordeirfcup/PLS-QSAR, accessed on 15 May 2023). These tools are intended to assist the scientific community in developing machine-learning-based regression models and PLS models, respectively.

## Figures and Tables

**Table 1:** List of descriptors of the 2D-QSAR model with their descriptions.

Name	Definition	Class
ATS6m	Broto-Moreau autocorrelation of lag 6 (log function) weighted by mass	2D Autocorrelation
J_Dz(p)	Balaban-like index from Barysz matrix weighted by polarizability	2D Matrix-based
CATS2D_07_AA	CATS2D Acceptor-Acceptor at lag 07	2D Pharmacophore
CATS2D_03_NL	CATS2D Negative-Lipophilic at lag 03	2D Pharmacophore
CATS2D_05_AA	CATS2D Acceptor-Acceptor at lag 05	2D Pharmacophore
SM14_AEA(dm)	Spectral moment of order 14 from augmented edge adjacency matrix weighted by the dipole moment	Edge adjacency indices
F09[N-O]	Frequency of N–O at topological distance 9	2D atom-pairs
RDF140v	Radial Distribution Function at a distance of 14.0 Å weighted by van der Waals volume	3D (RDF)

**Table 2:** Summary of the results obtained from non-linear models.

Descriptors	ML	*Q*^2^_LOO_ (5-fold)	*R* ^2^_Pred_	Average	Selected Parameters *
Linear model	MLP	0.767	0.797	0.780	activation = Identity, solver = Lbfgs, hidden layer Sizes = (5)
Linear model	RF	0.673	0.741	0.707	max_depth = 10, max features = Sqrt, min samples leaf = 2
Linear model	SVM	0.757	0.805	0.781	gamma = 1.0, kernel = Linear
dSe	MLP	0.391	0.632	0.442	activation = Identity, solver = lbfgs, hidden layer Sizes = (5)
dSe	RF	0.531	0.601	0.566	criterion: MAE, maximum depth = 30, max_features = Sqrt, n_estimators = 200
dSe	SVM	0.405	0.626	0.516	C = 100.0, gamma = 1.0, kernel = Linear

* If not mentioned, the default parameters were selected from the following links: kNN: https://scikit-learn.org/stable/modules/generated/sklearn.neighbors.KNeighborsRegressor.html accessed on 1 May 2023; MLP: https://scikit-learn.org/stable/modules/generated/ sklearn.neural_network.MLPRegressor.html accessed on 1 May 2023; RF: https://scikit-learn.org/stable/modules/generated/sklearn.ensemble.Random ForestRegressor.html accessed on 1 May 2023; SVM: https://scikit-learn.org/stable/modules/generated/sklearn.svm.SVR.html accessed on 1 May 2023. Whenever applicable, the models were generated using a random_state = 42.

**Table 3:** Results for the 3D-QSAR models with three components.

Parameter	FFD-SEL	UVE-PLS
*N*_training_	148	148
*R* ^2^	0.756	0.778
*F*	148.89	168.68
*Q* ^2^_LOO_	0.615	0.643
*Q* ^2^_LTO_	0.614	0.643
*Q* ^2^_LMO_	0.603	0.631
*N*_test_	36	36
*R* ^2^_Pred_	0.631	0.657

**Table 4:** MM-GBSA binding free energies (in kcal/mol) calculated for **S74**, **D4_02**, and **D2_37**.

Compound	ΔE_vdW_	ΔE_elec_	ΔG_polar_	ΔG_nonpolar_	TΔS	ΔG_bind_(T) ^a^
S74	−65.26	21.92	−0.43	−8.33	−28.43	−23.67
D4_02	−64.85	−27.18	71.00	−8.25	−12.43	−16.85
D2_37	−42.49	53.17	−32.44	−5.07	−27	0.17

^a^ ΔG_bind(T)_: theoretical binding free energy (=ΔE_vdW_ + ΔE_ele_ + ΔG_polar_ + ΔG_nonpolar_ − TΔS) and its components. ΔE_vdW_: van der Waals interaction energy; ΔE_ele_: electrostatic interaction energy; ΔG_polar_: polar solvation free energy; ΔG_nonpolar_: nonpolar solvation free energy; TΔS: entropy.

## Data Availability

Further details about the data presented in this study are available on request from the corresponding authors.

## Supplementary Materials

The following supporting information can be downloaded at: https://www.mdpi.com/article/10.3390/molecules28176379/s1, Figure S1. The cluster analyses on the t-SNE plot to depict the chemical space of the dataset compounds. Figure S2. The relevance of each atom of D1_01 (left) and D2_37 (right) as per the Layer-wise Relevance Propagation (LRP) algorithm as implemented in Transformer-CNN. Figure S3. Electrostatic maps of (A) D5_32 and (B) D4_02. Figure S4. PoseView (https://proteins.plus/; accessed on 3 June 2023) 2D diagram of the pose obtained from the final trajectory of the MD simulations for D4_02; Table S1: Details of the dataset used in this work; Table S2: Hyperparameters that were optimized in the development of non-linear models; Table S3: Summary of the linear 2D-QSAR models generated with different descriptor-calculating tools and feature selection techniques. Table S4: Correlation matrix for the 2D-QSAR model.

## Sample Availability

All data generated or analyzed during this study are included in this published article (and its supporting data files).
