# Supplementary material for: In Silico Modeling and Structural Analysis of Soluble Epoxide Hydrolase Inhibitors for Enhanced Therapeutic Design
Source: Molecules. 2023 Aug 31;28(17):6379. doi: 10.3390/molecules28176379 (PMC10490281; doi:10.3390/molecules28176379)
Supplement: Supplementary file 1 [file molecules-28-06379-s001.zip › TableS2S4_FigureS1S4.pdf]

Table S2. Hyperparameters which were optimized in the development of non-linear models

Machine learning Method	Parameters optimized
Multilayer perception (MLP)	hidden_layer_sizes=(5,), (10,), (50,), (100), (5,5,), (10,5,), (5,5,5,), (10,5,5,), (25,25,), (50,50,), etc.<br>activation: identity, logistic, tanh,relu<br>learning_rate: constant, adaptive, invscaling<br>solver: sgd, adam, lbfgs<br>alpha: 0.0001, 0.001, 0.01, 0.1
Random forest (RF)	criterion: mse, mae<br>max_features: auto, sqrt,log2<br>max_depth: 10,30,50,70,90,100,200<br>n_estimators: 5,10,20,50,100,200<br>min_samples_leaf: 1,2,4<br>min_samples_split: 2,5,10
Support vector machine (SVM)	C: 0.1,1,10,100,1000<br>gamma: 1,0.1,0.01<br>kernel: rbf, linear

Table S3. Summary of the linear 2D-QSAR models generated with different descriptor-calculating tools and feature selection techniques.

**SN**	**Descriptor**	**Method**	**Score**	**CV**	Q^2_{LOO}	R^2_{Pred}	**Average**
01	AlvaDes	SFS-MLR	R^2	0	0.788	0.767	0.778
02	AlvaDes	SFS-MLR	R^2	5	0.752	0.595	0.673
03	AlvaDes	SFS-MLR	NMAE	0	0.775	0.647	0.711
04	AlvaDes	SFS-MLR	NMAE	5	0.776	0.577	0.677
05	AlvaDes	SFS-MLR	NMPD	0	0.790	0.705	0.747
06	AlvaDes	SFS-MLR	NMPD	5	0.736	0.720	0.728
07	AlvaDes	SFS-MLR	NMGD	0	0.790	0.705	0.747
08	AlvaDes	SFS-MLR	NMGD	5	0.746	0.738	0.742
09	AlvaDes	GA-MLR	NA	NA	0.784	0.792	0.788
10	CDK	SFS-MLR	R^2	0	0.624	0.626	0.625
11	CDK	SFS-MLR	R^2	5	0.674	0.648	0.661
12	CDK	SFS-MLR	NMAE	0	0.666	0.617	0.641
13	CDK	SFS-MLR	NMAE	5	0.683	0.567	0.625

14	CDK	SFS-MLR	NMPD	0	0.611	0.634	0.623
15	CDK	SFS-MLR	NMPD	5	0.625	0.627	0.626
16	CDK	SFS-MLR	NMGD	0	0.611	0.634	0.623
17	CDK	SFS-MLR	NMGD	5	0.625	0.627	0.626
18	CDK	GA-MLR	NA	NA	0.690	0.662	0.676
19	GSFRAG+ISIDA	SFS-MLR	R^2	0	0.741	0.651	0.696
20	GSFRAG+ISIDA	SFS-MLR	R^2	5	0.746	0.610	0.678
21	GSFRAG+ISIDA	SFS-MLR	NMAE	0	0.723	0.600	0.661
22	GSFRAG+ISIDA	SFS-MLR	NMAE	5	0.716	0.587	0.652
23	GSFRAG+ISIDA	SFS-MLR	NMPD	0	0.732	0.657	0.695
24	GSFRAG+ISIDA	SFS-MLR	NMPD	5	0.743	0.632	0.688
25	GSFRAG+ISIDA	SFS-MLR	NMGD	0	0.732	0.657	0.695
26	GSFRAG+ISIDA	SFS-MLR	NMGD	5	0.714	0.528	0.621
27	GSFRAG+ISIDA	GA-MLR	NA	NA	0.723	0.677	0.700
28	Mordred	SFS-MLR	R^2	0	0.765	0.674	0.720
29	Mordred	SFS-MLR	R^2	5	0.746	0.581	0.663
30	Mordred	SFS-MLR	NMAE	0	0.734	0.583	0.658
31	Mordred	SFS-MLR	NMAE	5	0.705	0.598	0.652
32	Mordred	SFS-MLR	NMPD	0	0.751	0.600	0.675
33	Mordred	SFS-MLR	NMPD	5	0.743	0.603	0.673
34	Mordred	SFS-MLR	NMGD	0	0.761	0.630	0.695
35	Mordred	SFS-MLR	NMGD	5	0.747	0.637	0.692
36	Mordred	GA-MLR	NA	NA	0.746	0.730	0.738
37	MNA	SFS-MLR	R^2	0	0.773	0.560	0.667
38	MNA	SFS-MLR	R^2	5	0.759	0.600	0.679
39	MNA	SFS-MLR	NMAE	0	0.749	0.579	0.664
40	MNA	SFS-MLR	NMAE	5	0.744	0.642	0.693
41	MNA	SFS-MLR	NMPD	0	0.750	0.522	0.636
42	MNA	SFS-MLR	NMPD	5	0.762	0.521	0.642
43	MNA	SFS-MLR	NMGD	0	0.750	0.522	0.636
44	MNA	SFS-MLR	NMGD	5	0.760	0.529	0.645
45	MNA	GA-MLR	NA	NA	0.767	0.625	0.696
46	SIRMS	SFS-MLR	R^2	0	0.741	0.530	0.636
47	SIRMS	SFS-MLR	R^2	5	0.741	0.530	0.636
48	SIRMS	SFS-MLR	NMAE	0	0.734	0.127	0.430
49	SIRMS	SFS-MLR	NMAE	5	0.734	0.127	0.430
50	SIRMS	SFS-MLR	NMPD	0	0.738	0.372	0.555
51	SIRMS	SFS-MLR	NMPD	5	0.738	0.372	0.555

52	SIRMS	SFS-MLR	NMGD	0	0.728	0.403	0.566
53	SIRMS	SFS-MLR	NMGD	5	0.728	0.403	0.566
54	SIRMS	GA-MLR	NA	NA	0.717	0.622	0.670
55	MERA+MERSY	SFS-MLR	R^2	0	0.708	0.658	0.683
56	MERA+MERSY	SFS-MLR	R^2	5	0.724	0.708	0.716
57	MERA+MERSY	SFS-MLR	NMAE	0	0.668	0.603	0.636
58	MERA+MERSY	SFS-MLR	NMAE	5	0.664	0.616	0.640
59	MERA+MERSY	SFS-MLR	NMPD	0	0.648	0.646	0.647
60	MERA+MERSY	SFS-MLR	NMPD	5	0.696	0.666	0.681
61	MERA+MERSY	SFS-MLR	NMGD	0	0.709	0.636	0.672
62	MERA+MERSY	SFS-MLR	NMGD	5	0.699	0.596	0.647
63	MERA+MERSY	GA-MLR	NA	NA	0.683	0.716	0.699
64	RDKit	SFS-MLR	R^2	0	0.816	0.551	0.683
65	RDKit	SFS-MLR	R^2	5	0.824	0.504	0.664
66	RDKit	SFS-MLR	NMAE	0	0.811	0.550	0.681
67	RDKit	SFS-MLR	NMAE	5	0.816	0.458	0.637
68	RDKit	SFS-MLR	NMPD	0	0.816	0.551	0.683
69	RDKit	SFS-MLR	NMPD	5	0.804	0.559	0.681
70	RDKit	SFS-MLR	NMGD	0	0.792	0.570	0.681
71	RDKit	SFS-MLR	NMGD	5	0.804	0.559	0.681
72	RDKit	GA-MLR	NA	NA	0.758	0.670	0.714
73	PyDescriptors	SFS-MLR	R^2	0	0.780	0.519	0.650
74	PyDescriptors	SFS-MLR	R^2	5	0.763	0.418	0.591
75	PyDescriptors	SFS-MLR	NMAE	0	0.782	0.526	0.654
76	PyDescriptors	SFS-MLR	NMAE	5	0.762	0.496	0.629
77	PyDescriptors	SFS-MLR	NMPD	0	0.708	0.638	0.673
78	PyDescriptors	SFS-MLR	NMPD	5	0.66	0.589	0.625
79	PyDescriptors	SFS-MLR	NMGD	0	0.782	0.526	0.654
80	PyDescriptors	SFS-MLR	NMGD	5	0.666	0.586	0.626
81	PyDescriptors	GA-MLR	NA	NA	0.808	0.66	0.734

Table S4. Correlation matrix for the 2D-QSAR model.

Descriptors	ATS6m	F09[N-O]	CATS2D_05_AA	SM14_AEA(dm)	CATS2D_03_NL	RDF140v	CATS2D_07_AA	J_Dz(p)
ATS6m	1.00	-0.14	-0.03	0.74	-0.31	0.49	0.20	-0.62
F09[N-O]		1.00	-0.20	-0.05	0.16	-0.13	-0.19	0.11
CATS2D_05_AA			1.00	-0.29	-0.10	-0.28	0.03	0.33
SM14_AEA(dm)				1.00	-0.19	0.54	0.20	-0.75
CATS2D_03_NL					1.00	-0.28	-0.03	0.22
RDF140v						1.00	-0.04	-0.58
CATS2D_07_AA							1.00	-0.21
J_Dz(p)								1.00

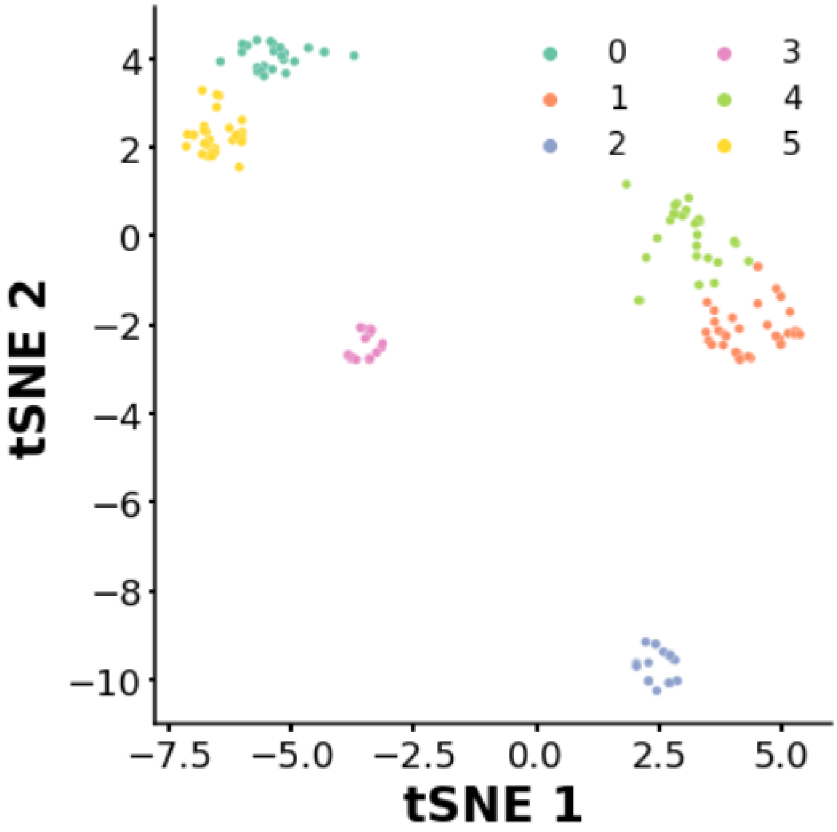

Figure S1. The cluster analyses on the t-SNE plot to depict the chemical space of the dataset compounds.

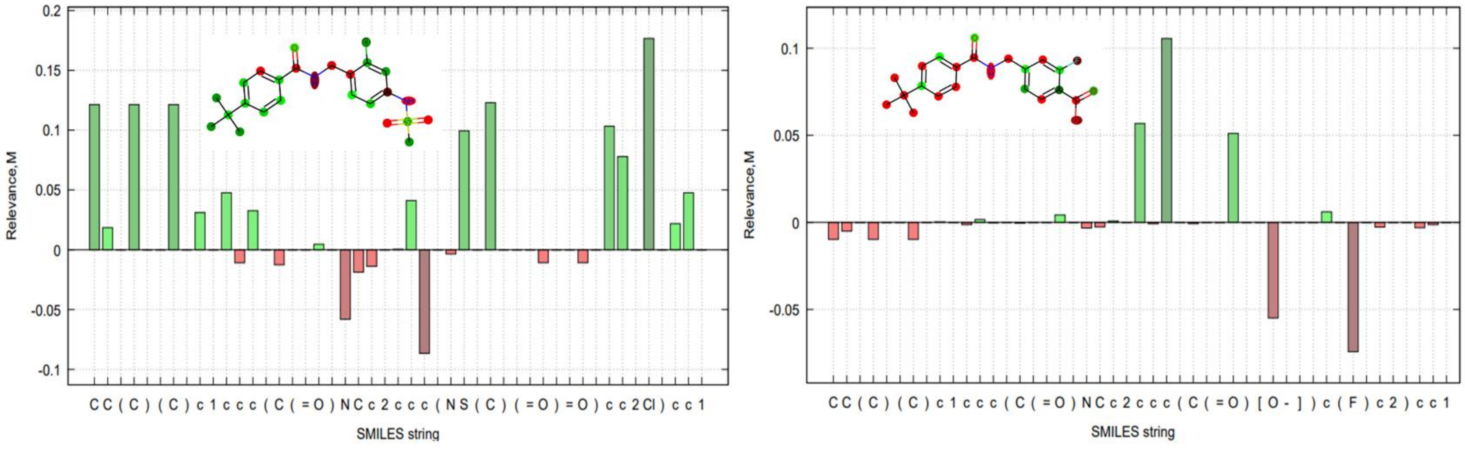

Figure S2. The relevance of each atom of **D1_01** (left) and **D2_37** (right) as per Layer-wise Relevance Propagation (LRP) algorithm as implemented in Transformer-CNN.

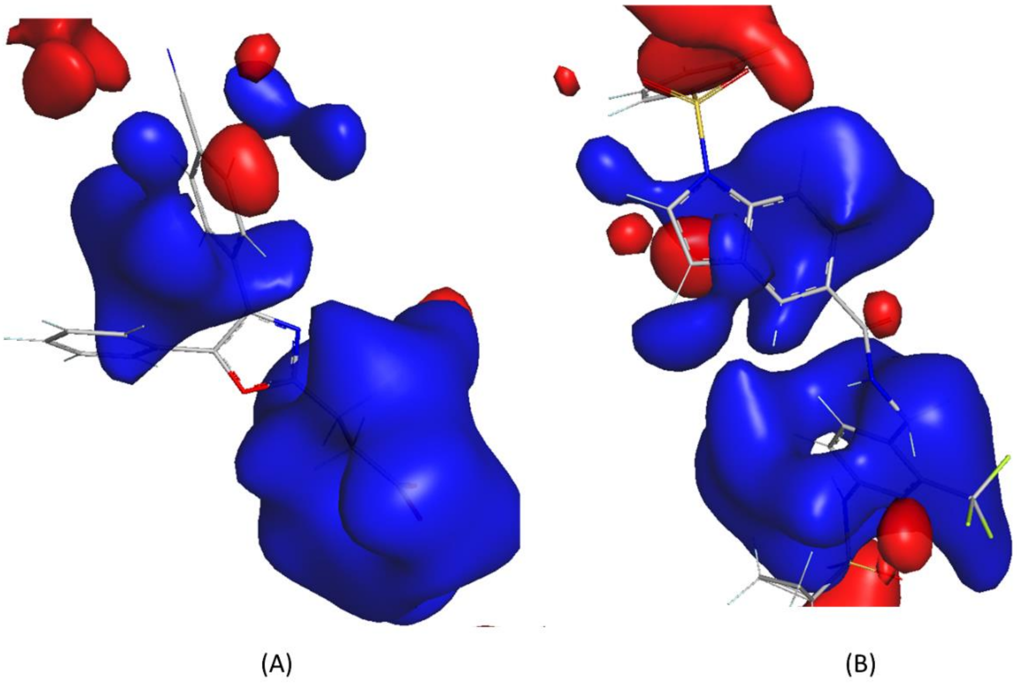

Figure S3. Electrostatic maps of (A) **D5_32** and (B) **D4_02**.

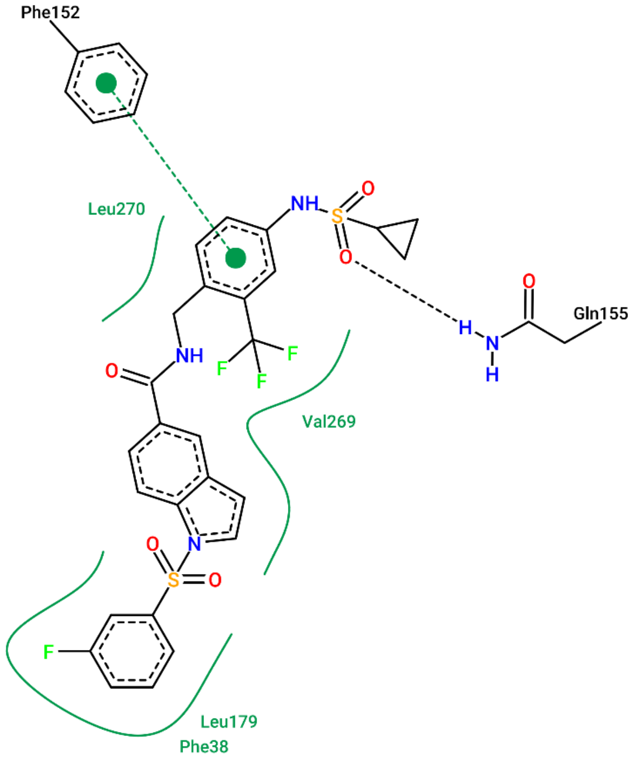

Figure S4. PoseView (https://proteins.plus/) 2D diagram of the pose obtained from the final trajectory of the MD simulations for **D4_02.**
